# A cross-sectional study on the perceptions and practices of modern and traditional health practitioners about traditional medicine in Dembia district, north western Ethiopia

**DOI:** 10.4103/0973-1296.59962

**Published:** 2010-02-13

**Authors:** Muthuswamy Ragunathan, Hawi tadesse, Rebecca tujuba

**Affiliations:** *Department of Pharmacognosy, PGP College of Pharmacy, Namakal, Tamilnadu, India*; 1 *Department of Pharmacognosy School of Pharmacy, University of Gondar, Ethiopia*

**Keywords:** Dembia district, Ethiopia, perceptions and practices, traditional health practitioners, traditional medicine

## Abstract

A cross-sectional study pertaining to the practices and perceptions of modern and traditional health practitioners on Traditional Medicine (TM) was carried out from February 25 to April 4, 2008. The results of the study showed that almost all the practitioners in both systems expressed their willingness to collaborate among each other to promote the positive elements of TM. As traditional healing knowledge is still being handed over from one generation to the next, mainly through word of mouth, which will lead to distortion or a total demise of the original knowledge, this report indicates the urgency to document the same. Moreover, the report also implies the need for educating and training the practitioners of the two systems. More also has to be done to create a discussion forum for both modern and TM practitioners, to enable them to share their knowledge. Government support for promotion and development of TM should be considered as a goal to be seriously pursued. The government should also contribute by helping them financially and by arranging training and education for the improvement of the healthcare system given to the public.

## INTRODUCTION

Traditional and modern systems of medicine were developed by different philosophies. They look at health, diseases, and causes of diseases in different ways. These differences bring different attitudes ranging from complete rejection of TM by modern medical practitioners and of modern medicine by traditional medical practitioners to a parallel existence with little communication over patient care. Experience from many countries, such as those in South East Asia, suggest that integration of traditional and modern healthcare systems can solve much of the problems by providing the basic healthcare services for people in developing countries, particularly the undeserved majority. In these countries, both systems are equally developed and supplement each other toward achieving optimal healthcare coverage.[1,2]

Harmonization of traditional and modern medicine emphasizes the importance of respectful coexistence. Within the model of harmonization, there is a requirement to develop and hold a good understanding between traditional and modern medicine.[1] Many traditionally used medicinal plants contain pharmacologically active compounds used in the preparation of both traditional and modern medicines. Over 25% of the pharmaceutical preparations in the world and more than 50% in the USA contain plant-derived active principles.[2] At present, most of the phytoconstituents and plant extracts find their way into modern medicine to treat many critical diseases, and fill the gap between the contemporary system and traditional system of medicine. With this background, this survey has been conducted among traditional and modern medicine practitioners, to perceive their attitude toward the integration and co-recognition of both systems of medicine.

### Study area

The study was carried out in Dembia district, Koladiba town, in the North Gondar zone of the Amhara National Regional Government of the Federal Democratic Republic of Ethiopia. The Dembia district bordered the Yelay Armacheho district in the North, Lake Tana in the South, Gondar town and Gondar zuria districts toward the East, and the Chilga and Takusa districts in the west. The capital of the district Koladiba is located 35 km away from Gondar town and about 765 km away from Addis Ababa, the metropolitan of Ethiopia, in the North West direction. The district is comprised of highlands at an altitude of 2080 to 1740 meters, covers a total area of 127,000 sq. km, and Koladiba has a surface area of 7.4 sq. km. Within the district are five urban areas and 40 rural areas make up a number of villages. This district has an estimated population of about 334,519. Among these 169,274 are males and 165,245 females. The ethnic group distribution in the district shows a larger portion belonging to Amhara being 212,243, and 4,685 in Kemant, 337 in Tigray, and 50 in Oromo; the majority being orthodox Christians. The rest are followers of other religions, mainly Islam. The district has eight health centers and 29 health posts. The working language is Amharic.[3]

## MATERIALS AND METHODS

A cross-sectional study of perceptions and practices of modern and traditional health practitioners was carried out From February to April, 2008 by using the convenience sampling technique. A total of 23 modern health professionals (MHPs) and 19 traditional health practitioners (THPs) who were available in the district during the time of the investigation were involved in the study. The MHPs were met at their respective working places, while the THPs were approached depending on their reputation, based on information obtained from the community.

Two types of questionnaires — one for the modern and another for the traditional health care practitioners — were used. The structured questionnaires were prepared in English and provided to MHPs for self-administration, and were translated into Amharic for an in-depth interview with the THPs. The principal investigators did the data collection. The responses were recorded in specially designed forms containing the respondents' personal information, and their knowledge and practice about traditional medicine. The collected data was checked for its completeness, accuracy, and consistency every day, and any ambiguity or incompleteness seen was corrected as soon as possible before proceeding to the next. The data was analyzed electronically using the Statistical Package for Social Science (SPSS) program on a computer and was summarized and presented in frequency tables, bar-graphs, and a pie-chart.

### Ethical consideration

The study was carried out after being approved by the University of Gondar, School of Pharmacy. Prior to filling the questionnaires, the participants' consent was obtained and they were assured that their responses would be used only for research purposes; and the information given would be treated with utmost care and confidentiality.

## RESULTS

### Sociodemographic characteristics

Table 1 shows selected sociodemographic characteristics of the study subjects. From a total of 23 modern health professionals, 11 (47.8%) were males and 12 (52.2%) were females. Of these, 78.2% were 26-35 years of age with only three (13.0%) serving for ≥10years. The nurses/mid-wives accounted for 52.2%, health extension workers, 17.4%, laboratory technicians and druggists 13.0% each, and health assistants 4.3%. The vast majority, that is, 18 (78.3%) were orthodox Christians along with two (8.7%) Muslims and three (13.0%) Protestants. The gender distribution of traditional healers was 15 (78.9%) and four (21.1%) for males and females, respectively. The proportion of healers above 45 years of age was larger than those below 45 years of age. All the interviewed healers were Amhara by ethnicity; 14 (73.7%) orthodox Christians and five (26.3%) Muslims. The distribution of healers by type of practice was 10 (52.6%) herbalists, three (15.8%) bonesetters, one (5.3%) traditional birth attendant, and five (26.3%) exercising two or more of the practices. The majority, that is, 73.7% had served for ≥10 years. With regard to the level of education, the illiterates and those who could read and write accounted for 21.1% each, those that had religious education 10.5%, and those who had modern education at elementary or senior high school levels accounted for 42.1%, with one (5.3%) healer educated above grade 12.

**Table 1:** Sociodemographic characteristics of modern and traditional health practitioners, Kolladiba, 2008

Variables	Modern health practitioners(n = 23)	Traditional healers(n = 19)
Gender		
Male	11(47.8)	15(78.9)
Female	12(52.2)	4(21.1)
Age in years		
20-25	4(17.5)	2(10.6)
26-35	18(78.2)	1(5.3)
36-45	1(4.3)	4(21.1)
46-55	-	7(36.7)
≥56	-	5(26.3)
Ethnicity		
Amhara	23(100)	19(100)
Religion		
Orthodox christian	18(78.3)	14(73.7)
Muslim	2(8.7)	5(26.3)
Protestant	3(13.0)	-
Years of service		
0-9	20(87.0)	5(26.3)
>10	3(13.0)	14(73.7)
Modern health practitioners by qualification		
Health assistant	1(4.3)	-
Nurse/Mid-wife	12(52.2)	-
Druggist	3(13.0)	-
Lab technician	3(13.0)	-
Health extension worker	4(17.4)	-
Traditional health practitioners by type of practice		
Herbalists	-	10(52.6)
Bone setters	-	3(15.6)
Traditional birth attendants	-	1(5.3)
Two or more of the practices	-	5(26.3)
Educational status		
Illiterate	-	4(21.1)
Read and write	-	4(21.1)
Grade 1-6	-	3(15.8)
Grade 7-8	-	2(10.5)
Grade 9-12	-	3(15.8)
Above grade 12	-	1(5.3)
Religious education	-	2(10.5)

Numbers within parenthesis are percentages

### Perceptions and practices of modern health practitioners

A majority of modern health practitioners, 60.9%, believed in the importance of TM for maintaining sufficient healthcare service to the community. Among the practitioners, 18 (78.3%) encountered patients who came soon after visiting traditional healers for their present complaint; and three (13.0%) of them came across patients who were advised (referred) by traditional healers to take medication in health centers. For personal medical remedy, only eight (34.8%) of the MHPs had visited traditional healers at least once in their lifetime. This along with the result recorded for personal preference for modern healthcare service showed that 18 (78.3%) demonstrated that most of the modern health practitioners chose the modern medical system over the traditional 21 (91.3%). As far as the fully supported cooperation of modern and traditional health practitioners and the integration of the two systems, four (17.4%) of them mentioned their personal experience of working (collaborating) with THPs.

**Figure 1:**
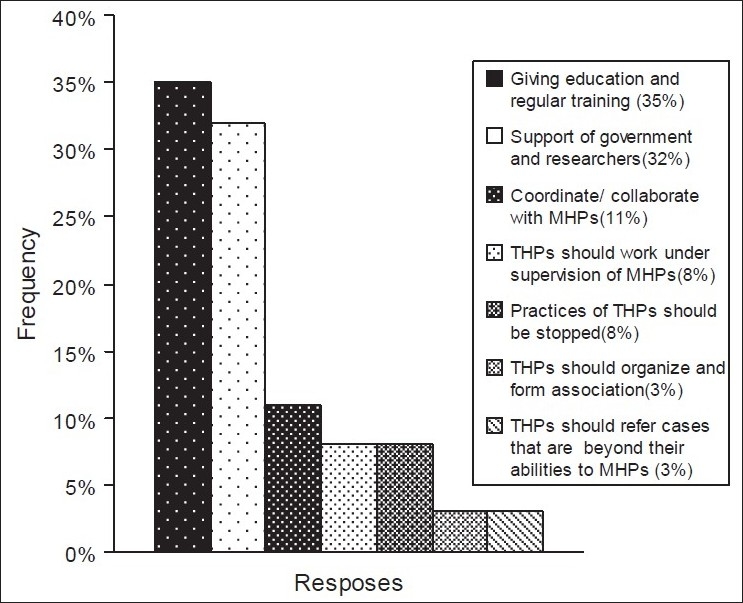
Solutions suggested by modern health professionals to improve traditional health practitioners

Almost all the MHPs 22 (95.7%) agreed to government support for THPs and the importance of scientific research into TM, for its promotion and development. They also sought different solutions they believed would bring about a change in the improvement of the practice. The majority, that is, 35% of the responses suggested educating and giving regular training to the THPs. Thirty-two percent of the responses indicated that government and/or researchers support to traditional practitioners would bring about the desired outcome. Coordinating and working of healers together with modern health professionals was the other suggestion made, which accounted for 11% followed by 8%, which implied it would be better if healers worked under the supervision of MHPs. Three percent of the responses recommended that healers became organized and formed an association among themselves. The same number of suggestions proposed that THPs refer serious cases that could go out of their hand to nearby health institutions, to be attended by MHPs. It might come as a surprise that another 8% of the responses suggested that traditional medical practices be totally stopped [Figure 1].

It was strongly felt the training of traditional healers was important for the improvement of the service. Twenty-one (91.3%) of the MHPs indicated the important areas on which traditional healers should be trained. Of these, 30.8% indicated training them on dose management, while 15.4% proposed training them on sterilization of equipments; 10.25% of the responses from the MHPs proposed that healers should be trained on the possible side effects of the medicaments they prepare. They also suggested training on proper and hygienic preparation of formulations, which accounted for 7.69% of the responses, even as, emphasis for their appropriate route of administration accounted for 7.69%. Of the total suggestions of the MHPs, 7.69% implied that THPs should be trained on the clean and proper management of wounds, so that the incidence of contamination was alleviated. Of the responses, 5.12% proposed that training on proper fracture healing should be given. Another 5.12% suggested that the traditional healers should be advised to refer high-risk cases to health institutions. Training of THPs in a particular area of specialization was also favored by the MHPs, accounting for 5.12% of the responses. Training on male circumcision was seen to account for 2.56% of the responses, while appropriate follow-up care suggested by MHPs constituted 2.56% of the responses [Table 2].

**Table 2:** Important areas of training traditional health practitioners suggested by modern health professionals

Particulars	Frequency n = 21 of the MHPs(%)
Dose management	30.8
Sterilization of equipment	15.4
Side effects of herbal medicine	10.25
Proper hygienic preparation of formulations	7.69
Correct and safe route administration	7.69
Clean and proper wound management	7.69
Fracture healing	5.12
Means of referring to health institution for extreme cases	5.12
Area of specialization in particular practice	5.12
Proper male circumcision	2.56
Careful follow-up and care	2.56

### Perceptions and practices of traditional healers

**Figure 2:**
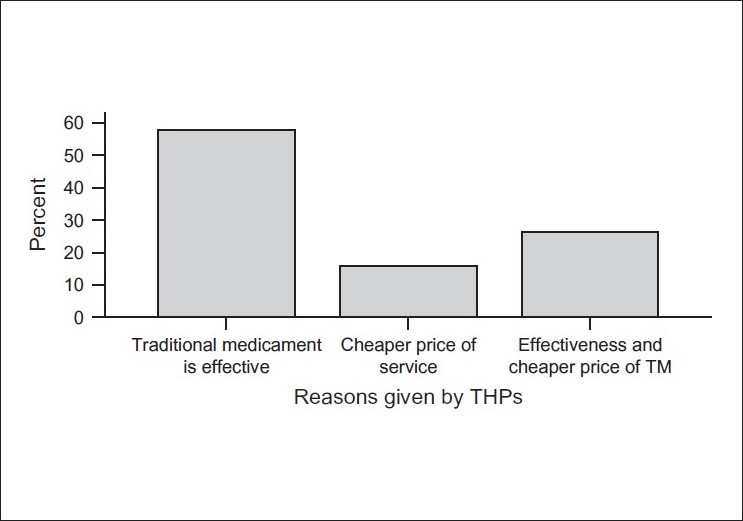
Reasons given by traditional health practitioners for the acceptance of traditional medicine by the community

Almost all THPs, 18 (94.7%), testified the acceptance of TM by the community. Only one (5.3%) healer refused to give an opinion about it. Of these THPs, 11 (57.9%) indicated the efficacy of the preparations as the main reason behind its popularity. This was followed by three (15.8%) who stated that acceptance was due to the cheaper price of the service, and five (26.3%) for the combination of the two reasons mentioned above [Figure 2].

The most frequently cited source of the knowledge of healing was from relatives, 14 (73.7%), followed by religious institutions, three (15.8%), and other healers, two (10.5%). Healers who were found to communicate (collaborate) with MHPs/researchers accounted for five (26.3%), while the other 73.7% had no collaboration with their counterparts in anyway. About 89.5% of the healers urged for training; 16 (84.2%) were completely willing to cooperate with the MHPs and supported integration of the two systems, to improve healthcare coverage in the country. According to all the respondents, none preferred to rely on modern healthcare services for seeking treatment, with six (31.6%) choosing to benefit from both systems, and 13 (68.4%) healers depending merely on traditional remedies [Table 3].

**Table 3:** Shows responses given by modern health practitioners and traditional healers, for selected questions that were common to both, regarding acceptance of traditional medical system, any collaboration with one another, integration of the two systems, about training of the traditional healers for the improvement of the practice, government/researchers support to traditional health practitioners and their personal preference of healthcare service

Question	MHP (n = 23) (%)	THP (n = 19) (%)
Do you accept traditional health care practice?		
Yes	14 (60.9)	-
No	9 (39.1)	-
Do you believe that the community accepts traditional medicine?		
Yes	-	18 (94.7)
No	-	1 (5.3)
Do you have any collaboration with THPs (for MHPs)/MHPs(for THPs)?		
Yes	4 (17.4)	5 (26.3)
No	19 (82.6)	14 (73.7)
Do you support cooperation of modern and traditional practitioners?		
Yes	21 (91.3)	16 (84.2)
No	2 (8.7)	3 (15.8)
Do you agree with the training of THPs for the improvement of the practice?		
Yes	21 (91.3)	17 (89.5)
No	2 (8.7)	2 (10.5)
Which healthcare service do you prefer personally?		
Modern	18 (78.3)	0 (0)
Traditional	1 (4.3)	13 (68.4)
Both	4 (17.4)	6 (31.6)

Half of the healers 10 (52.6%) responded to have collaboration with other traditional practitioners. Thirteen (68.4%) of them expressed their willingness to convey their knowledge, whereas, five (26.3%) preferred not to talk about their healing power, and one healer gave no opinion. Among the 19 THPs, 12 healers (63.2%) received the complete feedback from their patients. This is concerning patient's health status after the treatment provided. About six (31.6%) THPs collected only partial information from the patients whom they treated. Only one (5.3%) healer maintains a record of patient history, in which mentioned about the given modern drugs along with traditional medicaments. Regarding training on traditional medicine, none of the THPs, attended ever before. However, 16 (84.2%) of the THPs showed interest in taking training in the future if they getting the chance [Table 4].

**Table 4:** List of selected practices of traditional health practitioners

Variables	Frequency (n = 19) (%)
Traditional healers that have collaboration with other THPs	10 (52.6)
THPs that are willing to convey their knowledge	13 (68.45)
THPs that record history of their patients	1 (5.3)
THPs that look for full/partial information about the health status of their patients after providing treatment	18 (94.8)
THPs that provide modern drug(s) along with traditional medicaments	1 (5.3)
THPs that have taken training on TM before	0
THPs who would like to take training in the future	16 (84.2)

Numbers within parenthesis are percentages

**Figure 3:**
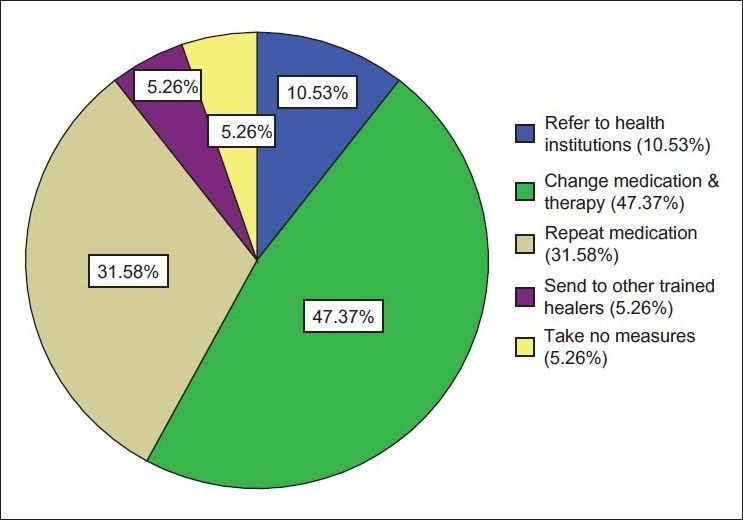
Measures taken by traditional health practitioners in cases of failure of their treatment

The healers were also asked about the failure of treatment they had provided and what they had done for their clients under such circumstances. They presented the different measures they had taken in those cases; 47.37% of the healers statedstated that they would change the medication and therapy, while 31.58% of the healers stated that they would go for a second trial by repeating the medication. About 10% of the healers statedstated that they would send their patients to MHPs if the treatment they provided could not help the client, while 5.26% of them stated that they would send the patient to another trained healer. Another 5.26% of the healers confessed that they would simply do nothing under such circumstances [Figure 3].

A majority of 13 (68.4%) healers stated that they did not have fixed payment cost for their service. They claimed that the rate and type of payment largely depended on the economic status of the patient. Fourteen (73.3%) of them rendered service free of charge to those who could not afford to pay. Of the six (31.6%) healers who had fixed cost for their service, the price range varied widely between ≥2 and ≤100 Ethiopian birr, with the maximum being not more than 30 birr. The average number of patients seen per week was reported to be five.

Out of 19 THPs, about 13 practitioners (68.4%) stated that there is no harmful effect from traditional health practice. This study also revealed about harmful practices of THPs. About 5 THPs (26.3%) approved Uvulectomy, and 3 THPs (15.8%) supported, tooth extraction, and 2 THPs (10.5%) accepted eyebrow cutting. This shows that majority of the THPs employed in one or more harmful traditional practices or at least they believed in the importance of such practices. The other six (31.6%) healers did not exercise any of these practices. The other finding that deserved appreciation was that all healers cursed (rejected) female circumcision, as it was not the culture of that society. Furthermore, 15 (78.9%) stressed the need for technical support from government/researchers.

As for the sources of medicaments, 17 (89.5%) of the healers stated that they mainly depended on plants, one (5.3%) on animal products, and one other healer used holy water for the treatment of ailments. Most of the healers, 14 (73.7%), stated that they largely made use of domestic plants followed by five (26.3%) who relied on both wild and domestic sources. According to the respondents, liquid (60.3%) was the most widely used dosage form followed by ointments 31.4%. They also added that the roots of the plants were most commonly used after the leaves. About 46.3% provided treatment for skin diseases, 20.1% for stomachache, parasite diseases, and diarrhea, 15.3% for sexually transmitted diseases, and 9.2% for TB and leprosy. Rabies, bone fracture, and others accounted for the other diseases treated by the THPs.

## DISCUSSION

Traditional Medicine has always been close to the large society, especially to those in developing countries, for meeting their primary healthcare needs. This popularity of TM has once again been proved by the present study, as it reveals that TM is widely practiced and accepted, particularly by the rural population.

It can be seen that over 75% of the traditional healers in our study group are males and this is in agreement with the previous reports.[4,5] The insignificant number of women in the interviewed healers may be because knowledge about the uses of medicinal plants and other traditional medical practices is mainly acquired from the family; and parents prefer to pass on their knowledge to their sons rather than their daughters. This also indicates that the knowledge is mainly transmitted through word of mouth, which will lead to distortion of the original knowledge or even bring about a total demise of the practice.

Taking into consideration the preference for a given healthcare delivery system, none of the healers favored modern medicine, as a great majority solely depends on the utilization of the traditional healthcare service. Similarly, most of the modern health practitioners preferred their own method of healthcare delivery system, which made it seem like everybody was in favor of what they were engaged in. However, this is not in agreement with a report from a pervious study conducted at the Arsi zone, on the same topic, where about 79% of the MHPs utilized TM and were in favor of TM even when conventional care was available.[1]

In the present study, about 95% of the healers believed that TM was accepted by their local communities. The main reason behind the preference of traditional to that of modern medicine was that the former was more efficacious than the latter. There were also other factors influencing the preference, such as, affordability, accessibility, and acceptability. These findings were synchronized with previous reports from the Arsi zone, Addis Ababa, and Ethiopia.[1,7] The proportion of THPs above 45 years of age was significantly larger than that of healers below 45 years of age. A similar finding was previously reported.[4] Perhaps, this could be one factor for the popularity of TM among local communities, as the elderly are respected and their principles are amenable.

Despite the common understanding about how difficult it would be for the two health systems to be integrated or how uncooperative the professionals would be to work together; our present finding demonstrated the existence of good will among most of the practitioners in both systems, for collaboration. Another study also confirmed that the alleged antagonism was an exaggeration.[5]

However, in actual practice, it is clear that the practitioners may not always reflect their orally expressed commitments. This can be proved by our documented result, wherein it was found that most of them did not interact with one another; and from a previous report[4] where more than 90% of the traditional healers stated they had no interaction with their modern counterparts.

Other than its positive contribution, the traditional healthcare system may also incorporate some harmful practices and beliefs.[6] It was unfortunate to come across a healer who adulterated TM with modern medicines and administered them to his patients. Furthermore, reports supporting this incidence indicated the possible existence of utilizing traditional medicaments along with pharmaceutical preparations by a significant number of individuals. Severe adverse effects have been reported for a number of herbal products from their indiscriminate adulteration by modern drugs. The direct consequences of such irresponsible drug use can lead to treatment failure, adverse effects, and antibiotic resistance. Unpredictable interactions between the medical herbs and modern drugs may take place, which could increase or decrease the pharmacological or toxicological effect of either or both components.[1]

Therefore, the traditional healers need to be educated in this aspect and patients should be advised against taking combined preparations. It was reported that some THPs have a fear of collaboration for the mere reason that they believe that if they reveal their methods of practice, it might not work for them again, other people might start practicing it as a result less number of patients will visit them or even patients might not come. Some also believe that they do not need more knowledge than they already have as they value the ones they got from their elders.

The Ethiopian community, in general, has the tendency for greater utilization of TM and thus, most of the THPs seek help from the government and are willing to get support. The MHPs on their part suggest certain areas of training, such as, sterilization of equipment, dosage management, clean and proper wound management, fracture healing, and in their total method of practice for improvement of TM.

## CONCLUSION

The study shows that none of the healers has been trained. As a result, improved relevant education is required for both sides. It is essential that researchers and MHPs be educated in both traditional and western medicines in order to perform research appropriately and treat patients effectively. Modern medical practitioners and researchers are required to achieve adequate education and awareness of the practice, principle, and context of TM. Similarly, THPs need to be significantly aware of the nature of practice and strengths of modern medical approaches. MHPs should help THPs in forming an association, leading to better organization among them, which would help in their cooperation with the MHPs. In this manner, both will develop goodwill for each other and consequently, will contribute to their collaboration. The government should also contribute by helping them financially and by arranging training and education, for the improvement of healthcare system given to the public.
